# A Ratiometric Fluorescent Probe for N_2_H_4_ Having a Large Detection Range Based upon Coumarin with Multiple Applications

**DOI:** 10.3390/molecules28227629

**Published:** 2023-11-16

**Authors:** Xiao Sheng, Xinfeng Sun, Yiwen Zhang, Chen Zhang, Shuling Liu, Shouxin Wang

**Affiliations:** School of Pharmaceutical Sciences, Jining Medical University, Rizhao 276826, China; shengxiao@mail.jnmc.edu.cn (X.S.); liushuling@mail.jnmc.edu.cn (S.L.)

**Keywords:** coumarin, hydrazine detection, ratiometric, fluorescent probe, cell imaging

## Abstract

Although hydrazine (N_2_H_4_) is a versatile chemical used in many applications, it is toxic, and its leakage may pose a threat to both human health and environments. Consequently, the monitoring of N_2_H_4_ is significant. This study reports a one-step synthesis for coumarin-based ratiometric fluorescent probe (FP) **CHAC**, with acetyl as the recognition group. Selected deprotection of the acetyl group via N_2_H_4_ released the coumarin fluorophore, which recovered the intramolecular charge transfer process, which caused a prominent fluorescent, ratiometric response. **CHAC** demonstrated the advantages of high selectivity, a strong capacity for anti-interference, a low limit of detection (LOD) (0.16 μM), a large linear detection range (0–500 μM), and a wide effective pH interval (6–12) in N_2_H_4_ detection. Furthermore, the probe enabled quantitative N_2_H_4_ verifications in environmental water specimens in addition to qualitative detection of N_2_H_4_ in various soils and of gaseous N_2_H_4_. Finally, the probe ratiometrically monitored N_2_H_4_ in living cells having low cytotoxicity.

## 1. Introduction

Hydrazine (N_2_H_4_) is a significantly active alkali as well as a reducing agent that has been widely applied in medicine, pesticides, materials, and dyes [1,2,3]. Furthermore, N_2_H_4_ is a well-known, high-energy fuel broadly employed in propulsion systems such as missiles and rockets [4,5]. However, N_2_H_4_ is a highly toxic substance, whose leakage in manufacturing, usage, transferring, and disposal can cause serious environmental problems. Furthermore, N_2_H_4_ can enter the human body via breathing and skin contact, thus leading to serious damage of the kidneys, liver, central nervous system, and lungs, which can also induce gene mutation or cancers [6,7,8]. The U.S. Environmental Protection Agency (EPA) has classified N_2_H_4_ as a candidate carcinogen with a 10 ppb (0.31 μM) threshold limit value [9]. Consequently, the development of simple and reliable methods that enable the detection of N_2_H_4_ trace amounts in the environment and living organisms is of utmost importance.

Several analysis technologies have been proposed to detect N_2_H_4_, such as chromatography [10], spectrophotometry [11], electrochemistry [12], potentiometry [13], and surface-enhanced Raman spectroscopy [14]. However, the majority of these technologies are costly, laborious, and require complex analytical procedures, greatly restricting their application to living biological specimens. Fluorescence-based methods, meanwhile, have been broadly applied to verify different analytes, including metal ions, anions, and biomolecules, because of their simple operation, high sensitivity, good selectivity, non-invasiveness, and good biocompatibility [15,16]. Several selective N_2_H_4_ fluorescent probes (FPs) with various recognition mechanisms [17,18], including selective deprotection [19,20,21,22]; reaction with diketones to form rings [23,24]; reactions with aromatic aldehydes, dicyanovinyl, or monocyanovinyl to form hydrazones [25,26,27,28,29]; cleavage of carbon–carbon double bonds to form hydrazones [30,31]; and hydrazine-induced ring-opening reactions [32,33] have been reported. However, many of these probes still possess disadvantages, such as their complex synthesis, narrow pH range, low sensitivity, and small detection range, which have limited their application. Furthermore, most hydrazine-based FPs adopt a fluorescence turn-on or turn-off strategy, and these single-emitting FPs are easily interfered with by probe concentrations, instrumental factors, and the test environment. Meanwhile, ratiometric FPs—based on ratios of emission intensity with two different wavelengths—can eliminate the possible external interference mentioned above and are thus more sensitive and accurate [34,35]. Consequently, an easy and reliable method allowing development of an effective ratiometric FP for N_2_H_4_ captures is of the utmost importance.

**Scheme 1:**
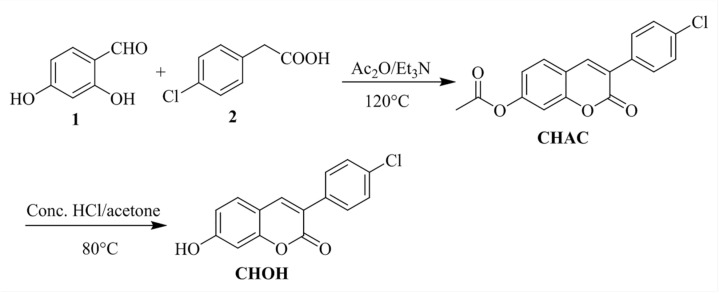
**CHAC** and **CHCO** synthetic routes.

Coumarins have been broadly utilized to develop FPs for distinguished analytes because of their high quantum yield, large Stokes shift, low cytotoxicity, and easy modification [36,37]. The introduction of reactive masking groups on the 7-hydroxylgroup of coumarin is an indispensable step in the design of coumarin-based N_2_H_4_ probes, whereby the masking groups are deprotected in the presence of N_2_H_4_, which causes significant fluorescence changes, mainly because of inhibition and the recovery of the intramolecular charge transfer (ICT) procedure concerning 7-hydroxycoumarin derivatives. Among these reported N_2_H_4_ probes based on 7-hydroxycoumarin, most of them belong to the turn-on variety [19,38,39,40,41,42,43,44,45,46,47,48], while ratiometric probes are rarely reported [49]. The current investigation prepared an ICT-based ratiometric FP **CHAC** for N_2_H_4_ detection via a simple, one-step reaction (Scheme 1). Although **CHAC** has been previously reported [50], as far as we know, this is the first time it has been used as an FP. The probe **CHAC** utilized a 7-hydroxycoumarin derivative **CHOH** as the fluorophore and an acetate ester as the recognition site. We deem that the selective ester cleavage in the probe by N_2_H_4_ will restore the push–pull electronic structure of **CHOH** and enhance the ICT effect, which will lead to a significant red shift with regard to the fluorescence emission spectra, thereby achieving ratiometric detection of N_2_H_4_. The experiment data showcased that **CHAC** exhibited perfect selectivity, a low detection limit (0.16 μM), a large detection range (0–500 μM), and a wide, effective pH range (6–12). Also, we successfully utilized **CHAC** to monitor N_2_H_4_ in environmental specimens as well as in living cells.

## 2. Results and Discussion

### 2.1. Design and Synthesis of the Probe CHAC

According to the design strategy of coumarin-based FPs [37], we introduced an electron-donating group (hydroxyl) at the 7-position and an electron-withdrawing group (4-chlorophenyl) at the 3-position of coumarin to form a push–pull π-conjugated system that could enhance the ICT effect, thereby resulting in an increase in fluorescence intensity and the emission wavelength of the fluorophore **CHOH**. The probe **CHAC** was prepared by introducing the recognition group acetyl to the hydroxyl group of **CHOH**. As an electron-withdrawing group, the acetyl group reduced the push–pull electron conjugation effect in **CHAC**. After reaction with N_2_H_4_, **CHAC** removed the acetyl group to release **CHOH** with a strong push–pull character, resulting in a significant red shift in the emission spectrum. As shown in Scheme 1, we prepared **CHAC** through a one-step condensation reaction. To investigate the response mechanisms of **CHAC** towards N_2_H_4_, the fluorophore **CHOH** was prepared via ester hydrolysis from **CHAC**. The **CHAC** and **CHOH** structures were verified through ^1^H NMR, ^13^C NMR and HRMS (Figures S1–S6).

### 2.2. Optical Properties

We tracked the UV-Vis and fluorescence spectra (FS) with regard to the probe **CHAC** for the absence and presence of N_2_H_4_ in DMSO:PBS buffer (10 mM, 4:6 *v*/*v*, pH = 7.4). The free probe (10 μM) showed obvious absorption at 327 nm (*ε* = 2.39 × 10^4^ M^−1^·cm^−1^) (Figure S7a). The addition of N_2_H_4_ (100 equiv) to the **CHAC** solution caused a decrement in the absorption peak at 327 nm accompanied by a novel band centered at 402 nm, which was consistent with that of **CHOH** (10 μM) (*ε* = 2.09 × 10^4^ M^−1^·cm^−1^). Upon excitation at 340 nm, the **CHAC** (5 μM) exhibited a single emission centered at 420 nm, which was red-shifted to 480 nm on its reaction with N_2_H_4_ (100 equiv), thus corresponding to a fluorescence color alternation from blue to cyan (Figure S7b). The resulting fluorescence spectrum was almost identical to that of **CHOH** (5 μM). We also measured the quantum yields (QYs) of **CHAC** and **CHOH** in the test solution, with quinine sulfate as the reference. The results show that both **CHAC** (*Φ* = 0.65) and **CHOH** (*Φ* = 0.46) produce high QYs. These data thus confirmed that **CHAC** could be utilized for fluorescent ratiometric detection of N_2_H_4_ via its transformation into **CHOH** mediated by N_2_H_4_.

### 2.3. Reaction Time and pH Effects

**Figure 1:**
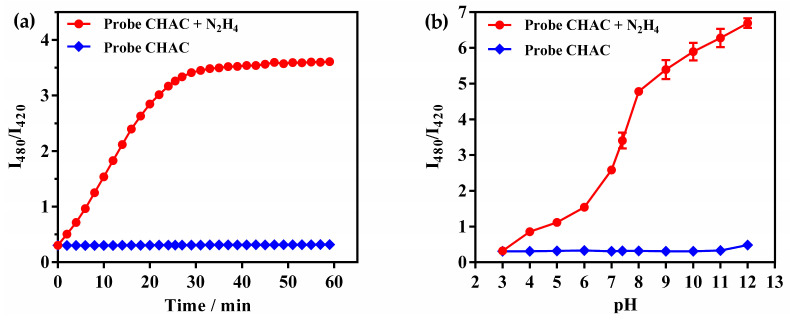
Time (**a**) and pH (**b**) effects upon the fluorescence intensity ratio (*I*_480_/*I*_420_) of **CHAC** (5 μM) in the absence and presence of N_2_H_4_ (500 μM).

The kinetics of the ratio responses of the probe **CHAC** to N_2_H_4_ and the stability of the probe itself were then investigated in DMSO:PBS buffer (10 mM, 4:6 *v*/*v*, pH = 7.4). In the absence of N_2_H_4_, the fluorescence intensity ratio (*I*_480_/*I*_420_) did not change during the testing duration, thus producing the inference that the probe was highly stable in the test solution under neutral conditions (Figure 1a). When 100 equiv of N_2_H_4_ was added, *I*_480_/*I*_420_ gradually increased as the reaction time increased, and a plateau was achieved within 30 min. Consequently, the reaction time was set to 30 min.

The pH effects upon N_2_H_4_ recognition by **CHAC** were investigated. In the absence of N_2_H_4_, the *I*_480_/*I*_420_ value did not change in pH ranges from 3 to 12, thus indicating that the free probe was very stable under acidic and alkaline conditions (Figure 1b). Upon incubating the probe with N_2_H_4_, *I*_480_/*I*_420_ gradually increased in the pH range of 3–12, and this increase was particularly significant in the pH range 6–12. This may be because alkaline conditions can prevent N_2_H_4_ from binding to hydrogen cations and also promote the hydrazinolysis of **CHAC**. Therefore, the probe **CHAC** was more suitable for the detection of N_2_H_4_ under neutral and alkaline conditions. A pH value of 7.4 was thus chosen to explore N_2_H_4_ in biological samples.

### 2.4. Fluorescence Spectra (FS) of Probes Titrated with N_2_H_4_

**Figure 2:**
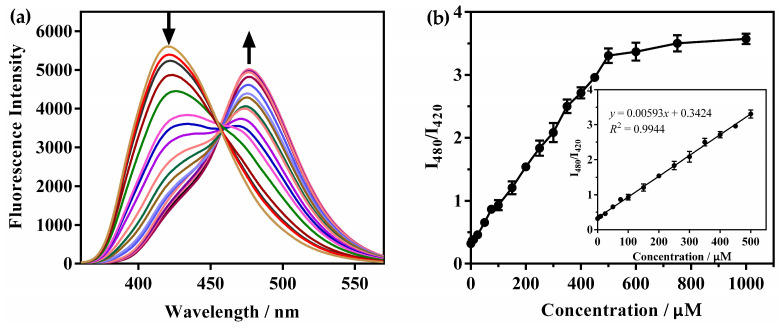
(**a**) **CHAC** (5 μM) FS after addition of various N_2_H_4_ concentrations (0–1000 μM) in DMSO:PBS buffer (10 mM, 4: 6 *v*/*v*, pH = 7.4); (**b**) plot of fluorescence intensity ratio *I*_480_/*I*_420_ versus the concentration of N_2_H_4_. Inset: The linearity of *I*_480_/*I*_420_ versus the concentration of N_2_H_4_ (0–500 μM).

The detection limit and linear range were determined via fluorescence titration of the probe **CHAC** with N_2_H_4_ (from 0 to 1000 μM). As the concentration of N_2_H_4_ increased, **CHAC** fluorescence emission at 480 nm gradually increased, while the fluorescence emission at 420 nm decreased markedly (Figure 2a). When the N_2_H_4_ concentration reached 500 μM, *I*_480_/*I*_420_ reached a plateau (Figure 2b). The plot of the *I*_480_/*I*_420_ ratio of **CHAC** versus N_2_H_4_ concentrations showcased a perfectly linear relationship (*R*^2^ = 0.9944) in the 0–500 μM ranges (inset of Figure 2b). The limit of detection (LOD) of **CHAC** was computed as 0.16 μM following 3*σ*/*K* (*σ*: the standard deviation of blank measurements; *K*: the linear regression line slope), which is less than the U.S. EPA standard (0.31 μM). Consequently, the **CHAC** probe demonstrates excellent sensitivity and can be used for the quantitative detection of N_2_H_4_. Compared to previously reported N_2_H_4_-based FPs (Table S1), **CHAC** features a large linear detection range, a low LOD, broad effective pH ranges, and versatile application in regard to N_2_H_4_, which makes **CHAC** more effective in detecting N_2_H_4_.

### 2.5. CHAC Selectivity and Anti-Interference Performance

**Figure 3:**
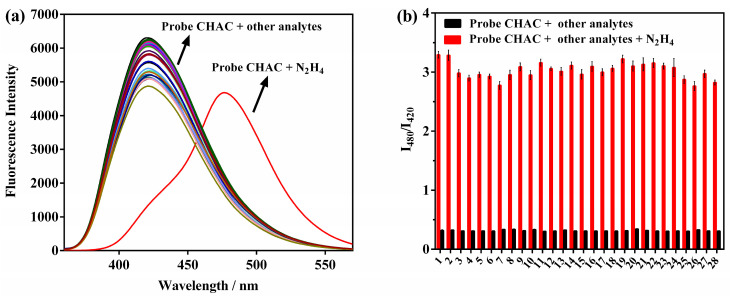
(**a**) **CHAC** (5 μM) FS upon N_2_H_4_ (500 μM) additions and different other analytes (500 μM) (1–28: Blank, Ammonia, Thiourea, Urea, NH_2_OH, Aniline, EDA, Cys, Hcy, GSH, CH_3_COO^−^, I^−^, $N_{3}^{-}$, F^−^, $H_{2}{\mathrm{PO}}_{4}^{-}$, ${\mathrm{SO}}_{4}^{2-}$, Cl^−^, Br^−^, ${\mathrm{HSO}}_{3}^{-}$, Fe^2+^, Ca^2+^, K^+^, Al^3+^, Mg^2+^, Na^+^, Fe^3+^, Cd^2+^, Zn^2+^); (**b**) Fluorescence intensity ratio *I*_480_/*I*_420_ of **CHAC** (5 μM) with various other analytes (500 μM), followed by 500 μM N_2_H_4_ addition.

To test **CHAC** selectivity for N_2_H_4_, we treated **CHAC** with various competitive analytes in a DMSO:PBS buffer (10 mM, 4:6 *v*/*v*, pH = 7.4). As illustrated in Figure 3a, when 100 equiv of related species—such as Cl^−^, Br^−^, F^−^, ${\mathrm{SO}}_{4}^{2-}$, I^−^, CH_3_COO^−^, $H_{2}{\mathrm{PO}}_{4}^{-}$, ${\mathrm{HSO}}_{3}^{-}$, $N_{3}^{-}$, K^+^, Na^+^, Ca^2+^, Mg^2+^, Al^3+^, Cd^2+^, Fe^3+^, Fe^2+^, Zn^2+^, ammonia, aniline, urea, thiourea, hydroxylamine, EDA, Cys, Hcy, or GSH—was incubated with **CHAC** (5 μM), no obvious variation in **CHAC** fluorescence characteristics was captured. However, adding 100 equiv N_2_H_4_ caused a significant shift in fluorescence emission from 420 to 480 nm. Furthermore, anti-interference experiments showed that the coexistence of all the relevant competing species mentioned above had few effects upon N_2_H_4_ detections by **CHAC** (Figure 3b). It was thus concluded that **CHAC** responds to N_2_H_4_ with perfect selectivity, and that the possibly coexisting interferential species did not interfere with this detection.

### 2.6. The Proposed Detection Mechanism

**Scheme 2:**
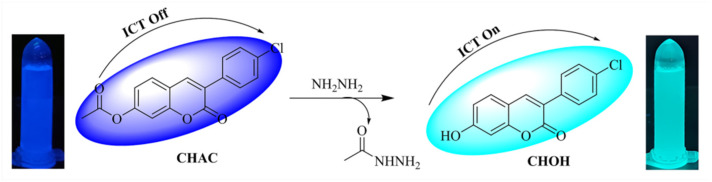
Proposed sensing mechanism of **CHAC** for N_2_H_4_.

**Figure 4:**
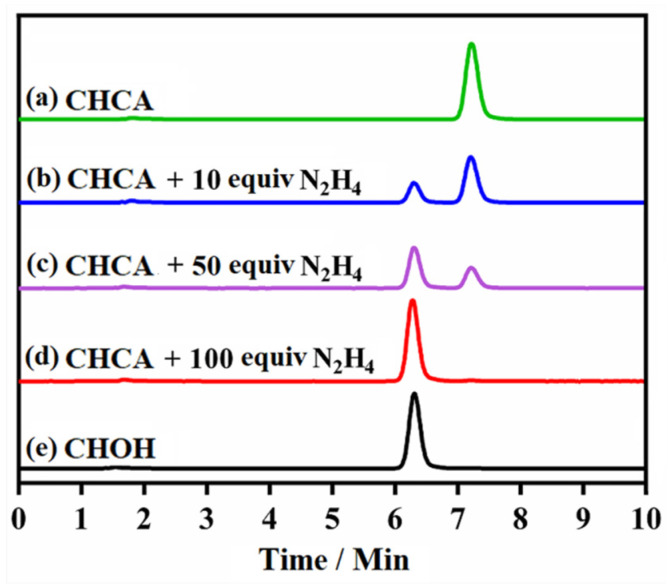
HPLC chromatograms of (**a**) probe **CHAC** (5 μM); (**b**) **CHAC** (5 μM) and 10 equiv N_2_H_4_; (**c**) probe **CHAC** (5 μM) and 50 equiv N_2_H_4_; (**d**) probe **CHAC** (5 μM) and 100 equiv N_2_H_4_; (**e**) compound **CHOH** (5 μM).

Based on the reported literature [38,40] and the results of UV-Vis and emission spectra (Figure S7), the probe **CHAC** response mechanism with N_2_H_4_ was proposed (Scheme 2). When a sufficient amount of N_2_H_4_ was added, it acted as a nucleophile to attack the acetoxy group in **CHAC**, and a hydrazinolysis reaction occurred that released the fluorophore **CHOH**. The proposed sensing process was further verified via HPLC and HRMS. From the HPLC chromatograms (Figure 4), the probe **CHAC** exhibited a main peak with a 7.22 min retention time. Upon post-incubation with N_2_H_4_, the **CHAC** peak gradually disappeared as the N_2_H_4_ concentration increased. A new peak also appeared at 6.31 min and gradually increased in intensity, which was attributed to **CHOH**. Furthermore, a main peak at *m*/*z* 271.0168 was discovered in the MS spectrum of the **CHAC** solution after the reaction with N_2_H_4_ (Figure S8), which was concordant with the deprotonated molecular weight of **CHOH** ([M–H]^−^ 271.0167). All these results indicated the conversion of **CHAC** into **CHOH** by N_2_H_4_.

**Figure 5:**
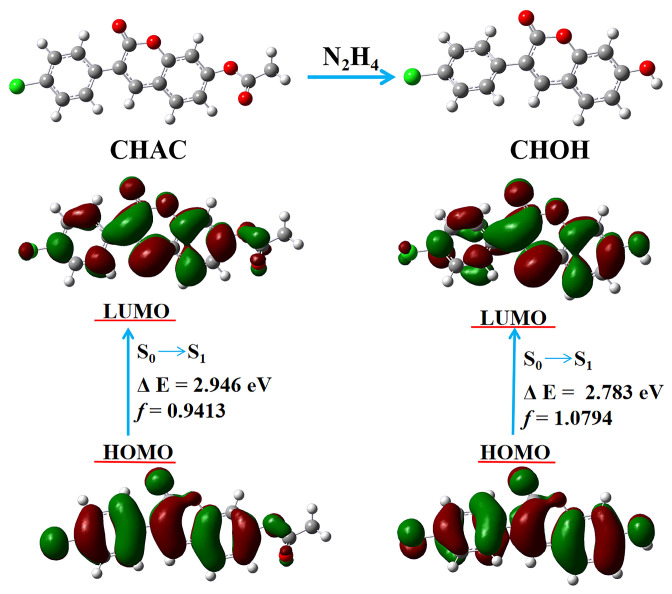
The energy diagrams of the optimized structures, LUMO, and HOMO of **CHAC** and **CHOH** were obtained from TDDFT calculations.

To help us understand the photophysical properties of probe **CHAC** before and after reaction with N_2_H_4_, we used the Gaussian 16 program [51] to perform time-dependent density functional theory (TDDFT) calculations at a B3LYP/6-311G(d,p) level. A polarizable continuum model (PCM) [52] with a dielectric constant of 78.54 was used to simulate a solvent environment for the calculation. As demonstrated in Figure 5, in the process of excitation from the ground state (S_0_) to the lowest excited state (S_1_), the probe **CHAC** and the fluorophore **CHOH** showed significantly different π-electron distributions. In the HOMO of **CHAC** and **CHOH**, the electron density was distributed evenly throughout the whole molecule, while that in the LUMO of these two molecules displayed a tendency to transfer to coumarin. Compared with the electron density in the LUMO of **CHOH**, which was found more locally on the pyranone ring of coumarin, that in the LUMO of **CHAC** was spread mainly over the entire coumarin, indicating that the acetyl group weakened the ICT process in the probe **CHAC**. In addition, the HOMO-LUMO gap of **CHOH** (2.783 eV) was slightly weaker compared with the probe **CHAC** (2.946 eV), which was concordant with the red shift of FS post-hydrazinolysis.

### 2.7. N_2_H_4_ Determinations for Water Samples

To verify the potential application of the probe we designed, we employed **CHAC** to capture N_2_H_4_ in water samples using the addition method. Tap and lake water were selected as representatives of actual water samples. The results revealed that the N_2_H_4_ concentrations detected by this probe were consistent with the added concentrations (Table 1). Recoveries for the determination of N_2_H_4_ were between 99.5% and 107.2%, thus indicating that **CHAC** can be utilized as a practical means for the detection of N_2_H_4_ in environmental specimens.

### 2.8. Gaseous N_2_H_4_ Detections

**Figure 6:**
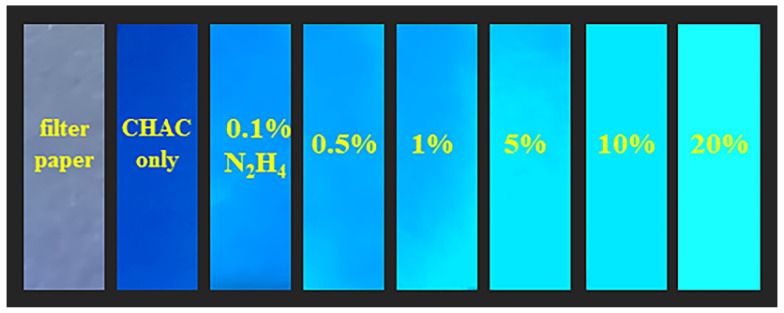
Figures of probe-coated filter papers on exposure to gaseous N_2_H_4_ from N_2_H_4_ aqueous solutions of various concentrations.

Another practical application of **CHAC** in N_2_H_4_ vapor capture was also investigated by loading **CHAC** onto filter paper. We pre-treated filter papers via the probe by immersing them in a methanol solution (1 mM) and then drying them. The filter papers were then placed in airtight jars that included distinguished N_2_H_4_ concentrations (0, 0.1%, 0.5%, 1%, 5%, 10%, and 20%), and were then exposed to N_2_H_4_ vapor for 0.5 h at room temperature. The results revealed that under a 365 nm light, N_2_H_4_ concentration increased and the fluorescent color of the filter paper gradually turned from deep blue to cyan (Figure 6). N_2_H_4_ vapor concentrations of 0–20% resulted in a noticeable difference in the **CHAC** color on the filter paper, which indicated that the probe **CHAC** could also be conveniently utilized for the qualitative detection of gaseous N_2_H_4_.

### 2.9. N_2_H_4_ Determinations in Soils

**Figure 7:**
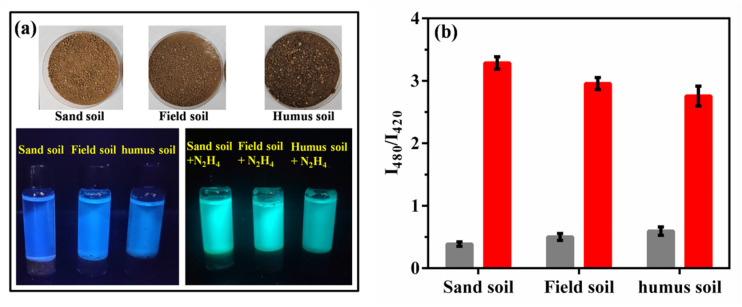
Photographs under 365 nm UV lamp (**a**) and fluorescence intensity ratio *I*_480_/*I*_420_ (**b**) of probe **CHAC** solution (5 μM) after the addition of untreated and N_2_H_4_ pretreated and soils.

Subsequently, the probe **CHAC** applications in soil samples were also explored [53]. Sandy, field, and humus soils were collected from the surrounding area as experimental samples. First, 1 g sandy soil/field soil/ humus soil was added to 3 mL of probe **CHAC** solution (5 μM). We discovered strong blue fluorescence under 365 nm UV light (Figure 7a), indicating that the soil itself did not influence the fluorescence properties of **CHAC**. Another 1 g of the abovementioned soil samples was first treated with N_2_H_4_ solution (2 mM), and then transferred to a solution of 3 mL **CHAC** (5 μM). After 30 min, the solution showed strong cyan fluorescence under 365 nm UV light. The supernatant of soil solution was then analyzed via fluorescence spectrometry. The data highlighted that the fluorescence intensity ratio *I*_480_/*I*_420_ of the soil solution treated with N_2_H_4_ was significantly higher than that without N_2_H_4_ (Figure 7b), which demonstrated that **CHAC** could be utilized for the ratiometric detection of N_2_H_4_ in soils.

### 2.10. Toxicity of Probe CHAC and Imaging of Cells

The cytotoxicity of the probe **CHAC** (10–50 μM) on MC3T3-E1 cells was evaluated using the MTT assay. These data revealed that, even after treatment with the 50 μM probe **CHAC** for 1 day, the cells maintained a viability of more than 90%, thus indicating the very low cytotoxicity of the probe **CHAC** (Figure S9).

**Figure 8:**
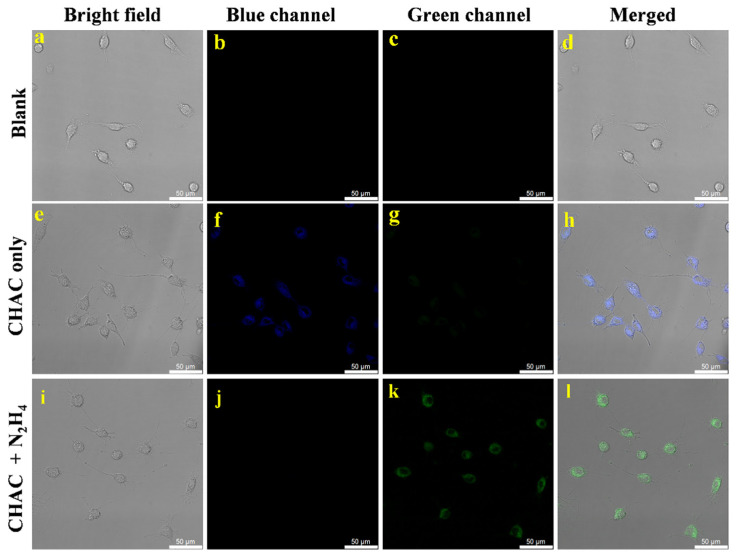
Confocal fluorescence images (FIs) in MC3T3-E1 cells. FI regarding MC3T3-E1 cells alone (**a**–**d**); Cells incubated through 10 μM **CHAC** for 0.5 h at 37 °C (**e**–**h**); Cells pretreated via 10 μM **CHAC** and incubated with 500 μM N_2_H_4_ for 0.5 h at 37 °C (**i**–**l**). *λ*_ex_ = 405 nm.

The imaging results of **CHAC** in MC3T3-E1 cells are shown in Figure 8. Under excitation at 405 nm, the cells showed fluorescence in neither blue nor green channels (Figure 8b,c). If we treated cells with **CHAC** alone for 0.5 h, strong fluorescence appeared in the blue channel, and almost no green fluorescence was found (Figure 8f,g). For cells pre-incubated with **CHAC** for 0.5 h and with N_2_H_4_ for another 0.5 h, the fluorescence in the blue channel disappeared, and strong fluorescence appeared in the green channel (Figure 8j,k), which indicated that the probe **CHAC** possessed effective cell membrane permeability to enable the fluorescent ratiometric detection of exogenous N_2_H_4_ in living cells.

## 3. Materials and Methods

### 3.1. Materials and Apparatus

2,4-Dihydroxybenzaldehyde, 4-chlorophenylacetic acid, triethylamine, 3-(4,5-dimethylthiazol-2-yl)-2,5-diphenyltetrazolium bromide (MTT), glutathione (GSH), cysteine (Cys), homocysteine (Hcy), and quinine sulfate were purchased from Shanghai Macklin Biochemical Co., Ltd (Shanghai, China). Acetic anhydride, ethylenediamine (EDA), aniline, urea, thiourea, hydroxylamine, N_2_H_4_, and various inorganic salts were purchased from Sinopharm Chemical Reagent Co., Ltd (Shanghai, China). Mouse embryonic osteoblasts (MC3T3-E1) were purchased from the National Experimental Cell Resource Sharing Platform (Beijing, China). All solvents used in the experiments were of commercially analytical or spectroscopic grade. Ultrapure, de-ionized water was utilized in the experiments.

NMR spectra were recorded on a Bruker Avance 400 MHz spectrometer (Bruker, Bremen, Germany), employing TMS as an internal reference. HRMS spectra were recorded using an Agilent 6540 Q-TOF spectrometer (Agilent, Santa Clara, CA, USA). UV-Vis spectra were measured using a Shimadzu UV-2450 spectrophotometer (Shimadzu, Kyoto, Japan). FS were recorded using a Hitachi F-4600 fluorescence spectrometer. pH values were determined utilizing a Leici PHSJ-4A acidometer (Leici, Shanghai, China). Cell imaging was performed using Leica Stellaris 5 confocal microscopy (Leica, Wetzler, Germany). Cytotoxicity tests were carried out on a DNM-9602G enzyme-labeled instrument (Perlong, Beijing, China). HPLC analysis was performed using a Shimadzu Prominence LC-20A liquid chromatograph (Shimadzu, Kyoto, Japan).

### 3.2. Synthesis of the Probe **CHAC**

2,4-Dihydroxybenzaldehyde (0.54 g, 3.61 mmol) and 4-chlorophenylacetic acid (0.93 g, 5.45 mmol) were dissolved in 8 mL acetic anhydride, and then 2 mL triethylamine was added. The mixture was heated to 120 °C and stirred for 7 h. The end of the reaction was monitored using TLC (EtOAc:petroleum ether = 1:3). After cooling, the white precipitate was collected, washed using water, dried, and then recrystallized with ethanol to obtain **CHAC** (0.81 g, yield 71%). ^1^H NMR (400 MHz, DMSO-*d*_6_): *δ* 8.31 (s, 1H), 7.82 (d, 1H, *J* = 5.4 Hz), 7.76 (d, 2H, *J* = 5.1 Hz), 7.54 (d, 2H, *J* = 5.1 Hz), 7.32 (s, 1H), 7.20 (d, 1H, *J* = 5.4 Hz), 2.32 (s, 3H). ^13^C NMR (100 MHz, DMSO-*d*_6_): *δ* 168.8, 159.4, 153.5, 152.8, 140.4, 133.4, 133.3, 130.3, 129.6, 128.3, 124.9, 118.9, 117.3, 109.7, 20.9. HRMS *m*/*z* calcd. for C_17_H_12_ClO_4_ [M+H]^+^: 315.0419, found: 315.0419.

### 3.3. Synthesis of the Fluorophore **CHOH**

**CHAC** (0.60 g, 1.91 mmol) was added to a mixture with 4 mL acetone and 8 mL concentrated hydrochloric acid, and the mixture was heated to 80 °C and stirred for 4 h. TLC (EtOAc:petroleum ether = 1:2) showed that **CHAC** was consumed. After cooling to room temperature, the yellow precipitate was collected, cleaned using water, and dried to collect **CHOH** (0.44 g, 85% yield). ^1^H NMR (400 MHz, DMSO-*d*_6_): *δ* 10.65 (s, 1H), 8.19 (s, 1H), 7.73 (d, 2H, *J* = 5.6 Hz), 7.68 (d, 1H, *J* = 5.7 Hz), 7.49 (d, 2H, *J* = 5.6 Hz), 6.83 (dd, 1H, *J* = 5.6 Hz, *J* = 1.0 Hz), 6.76 (s, 1H). ^13^C NMR (100 MHz, DMSO-*d*_6_): *δ* 161.5, 159.9, 155.0, 141.4, 133.9, 132.6, 130.1, 130.0, 128.2, 120.81, 113.5, 111.9, 101.7. HRMS *m*/*z* Calcd for C_15_H_9_ClNaO_3_ [M+Na]^+^: 295.0132, found: 295.0137.

### 3.4. Preparations for Spectral Measurement

The stock solution (5 mM) of **CHAC** was prepared in DMSO. Stock solutions (10 mM) of sodium salts (I^−^, F^−^, ${\mathrm{SO}}_{4}^{2-}$, ${\mathrm{HSO}}_{3}^{-}$, $H_{2}{\mathrm{PO}}_{4}^{-}$, Cl^−^, Br^−^, CH_3_COO, and $N_{3}^{-}$), cations (K^+^, Na^+^, Ca^2+^, Mg^2+^, Al^3+^, Cd^2+^, Fe^3+^, Fe^2+^, and Zn^2+^), and amino-containing analytes (ammonia, aniline, urea, thiourea, hydroxylamine, Cys, EDA, Hcy, and GSH) were obtained by dissolving them in deionized water. The test solutions were prepared as follows: **CHAC** (3 μL, 5 mM) and the analyte solution were added to a tube and diluted to 3 mL using DMSO: PBS buffer (10 mM, 4:6 *v*/*v*, pH = 7.4). We incubated the resulting solution for 0.5 h at room temperature and the fluorescence spectrum was recorded at an excitation wavelength of 340 nm. Emission and excitation slit widths were 5 nm and 2.5 nm, respectively, and the voltage was 700 V.

### 3.5. Determination of Quantum Yield (QY)

The QYs of the probes **CHAC** and **CHOH** were determined using quinine sulfate (*Φ* = 0.54 in 0.1 M H_2_SO_4_) as a reference. To obtain reliable data, the absorbance of all solutions should not be higher than 0.05. The QY was calculated using the following equation [54]:

(1) $$\Phi_{r}=\Phi_{s}\times \left(\frac{F_{S}}{F_{r}}\right)\times \;\left(\frac{A_{r}}{A_{s}}\right)\times \;{\left(\frac{n_{s}}{n_{r}}\right)}^{2}$$

where *Φ_r_* and *Φ_s_* represent the QY of the sample and the reference, respectively. *Fs* and *F_r_* denote the integral areas of the *Fs* of the sample and the reference under the same test conditions, respectively. *A_s_* and *A_r_* denote the absorbance of the sample and the reference, respectively. *n_s_* and *n_r_* indicate the refractive index of the sample and the reference in the corresponding solvent, respectively.

### 3.6. HPLC Detection

The test concentrations of both **CHAC** and **CHOH** were 5 μM. The injection volume was 10 μL. The mobile phase was CH_3_OH/H_2_O (7:3 *v*/*v*) and the flow rate was 1 mL/min. A Diamonsil C18(2) column (150 mm × 4.6 mm, 5 μm) was employed as the chromatographic column.

### 3.7. Detection of N_2_H_4_ in Water Samples

A standard addition approach was utilized to determine N_2_H_4_ content in water samples [55]. Our team obtained tap and lake water aliquots from our laboratory and Jian Lake, respectively. The water samples were filtered through a microporous membrane to remove impurities, and then replaced with a PBS buffer for fluorescence spectrometry. Various N_2_H_4_ concentrations were spiked into the water samples and subsequently analyzed using **CHAC**. Every group of experiments was performed simultaneously 3 times.

### 3.8. Cytotoxicity Test

We culturedMC3T3-E1cells in a DMEM containing 10% FBS at 37 °C under CO_2_ atmosphere (5%). Initially, we cultured MC3T3-E1 cells in 96-well plates for 1 day and allowed them to adhere. Different concentrations of **CHAC** (10, 20, 30, 40, and 50 μM) were then added and incubated for another 1 day. The culture media in the wells were removed and an MTT solution (5 mg/mL, 20 μL) was added to every well. Medium was then supplemented and incubated for an additional 4 h under the same conditions. After removing solution from the well, we added DMSO (150 μL/well) to dissolve the formed formazan precipitate. To calculate the cytotoxicity, we captured solution absorbance at 490 nm via a microplate reader.

### 3.9. Living Cell Fluorescence Imaging (FI)

MC3T3-E1 cells cultured under the aforementioned conditions were used for cell FI experiments. We incubated **CHAC** (10 μM) and MC3T3-E1 cells at 37 °C for 0.5 h, washed them 3 times with PBS buffer, and divided them into 2 groups. One group was directly imaged utilizing a fluorescence confocal microscope, while the other group of cells was treated with N_2_H_4_ (500 μM), incubated for an additional 0.5 h, and cleaned 3 times with PBS prior to imaging experiments. Blank MC3T3-E1 cells were used as the controls. We performed FI utilizing a confocal laser scanning microscope at 420–460 nm and 480–520 nm upon excitation at 405 nm.

## 4. Conclusions

We developed a new ratiometric N_2_H_4_ FP, **CHAC**, through a simple one-step reaction. The N_2_H_4_ presence produced a significant ratiometric alteration in the fluorescence emission curve by cleaving the acetate and releasing the coumarin fluorophore. The response mechanism of **CHAC** for N_2_H_4_ was validated through HRMS, HPLC, and TDDFT computations. **CHAC** exhibited high selectivity, strong anti-interference, a low LOD, a large detection range, and a broad pH application range. In addition, the **CHAC**-loaded filter paper strips could detect N_2_H_4_ vapor effectively via fluorescence color change. More importantly, we successfully employed **CHAC** for N_2_H_4_ detection in various water and soil specimens, as well as in ratiometric imaging regarding N_2_H_4_ in live cells with low toxicity. These data indicate that **CHAC** could be employed as a useful tool to capture N_2_H_4_ in environmental and biological systems.

## Figures and Tables

**Table 1:** N_2_H_4_ detection in water samples.

Sample	Added (μM)	Found (μM)	Recovery (%)	RSD (%)
Tap water	0	Not detected	—	—
	5.00	5.36	107.2	1.1
	25.00	25.51	102.0	0.8
	50.00	52.07	104.1	3.6
Lake water	0	Not detected	—	—
	5.00	5.13	102.2	2.8
	25.00	24.91	99.5	0.8
	50.00	51.02	102.0	3.3

## Data Availability

Data are contained within the article and Supplementary Materials.

## Supplementary Materials

The following supporting information can be downloaded at: https://www.mdpi.com/article/10.3390/molecules28227629/s1, Figures S1 and S4: ^1^H NMR spectrum of **CHAC** and **CHOH**; Figures S2 and S5: ^13^C NMR spectrum of **CHAC** and **CHOH**; Figures S3 and S6: HRMS spectrum of **CHAC** and **CHOH**. Figure S7: UV–Vis absorption (a) and fluorescence emission (b) spectra of **CHOH** (10 μM and 5 μM), and **CHAC** (10 μM and 5 μM) pre- and post-reaction with N_2_H_4_ (100 equiv) in DMSO:PBS buffer (10 mM, 4:6 *v*/*v*, pH = 7.4), *λ*_ex_ = 340 nm. Figure S8: HRMS spectrum of **CHAC** after reaction with N_2_H_4_. Figure S9: Various **CHAC** concentrations and their effects upon MC3T3-E1 cell viability. Table S1: Comparison of **CHAC** and other probes for N_2_H_4_. References [56,57,58,59,60,61,62,63,64,65] are cited in Supplementary Materials.
