# Supplementary material for: A Ratiometric Fluorescent Probe for N2H4 Having a Large Detection Range Based upon Coumarin with Multiple Applications
Source: Molecules. 2023 Nov 16;28(22):7629. doi: 10.3390/molecules28227629 (PMC10674487; doi:10.3390/molecules28227629)
Supplement: Supplementary file 1 [file molecules-28-07629-s001.zip › molecules-2683955-supplementary.pdf]

A Ratiometric Fluorescent Probe for N_2H_4 Having Large Detection Range Based upon Coumarin with Multiple Applications

Xiao Sheng, Xinfeng Sun, Yiwen Zhang, Chen Zhang, Shuling Liu and Shouxin Wang *

School of Pharmaceutical Sciences, Jining Medical University, Rizhao 276826, China

* Correspondence: shouxinwang@mail.jnmc.edu.cn

Table of contents

Figure S1. ^{1}H NMR spectrum of **CHAC** (400 MHz, DMSO-d_6)

Figure S2. ^{13}C NMR spectrum of **CHAC** (400 MHz, DMSO-d_6)

Figure S3. HRMS spectrum of **CHAC**

Figure S4. ^{1}H NMR spectrum of **CHOH** (400 MHz, DMSO-d_6)

Figure S5. ^{13}C NMR spectrum of **CHOH** (400 MHz, DMSO-d_6)

Figure S6. HRMS spectrum of **CHOH**

Figure S7. UV–Vis absorption (a) and fluorescence emission (b) spectra of **CHOH** (10 μM and 5 μM), and **CHAC** (10 μM and 5 μM) in piror and post reacting with N_2H_4 (100 equiv) in DMSO: PBS buffer (10 mM, 4:6 v/v, pH = 7.4), λ_{ex}= 340 nm. The inset of (b) shows photographs of **CHAC** with or without hydrazine and **CHOH** under UV light.

Figure S8. HRMS spectrum of **CHAC** after reaction with N_2H_4

Figure S9. Various **CHAC** concentrations effects upon MC3T3-E1 cell viability

Table S1. Comparison regarding **CHAC** and other probes for N_2H_4

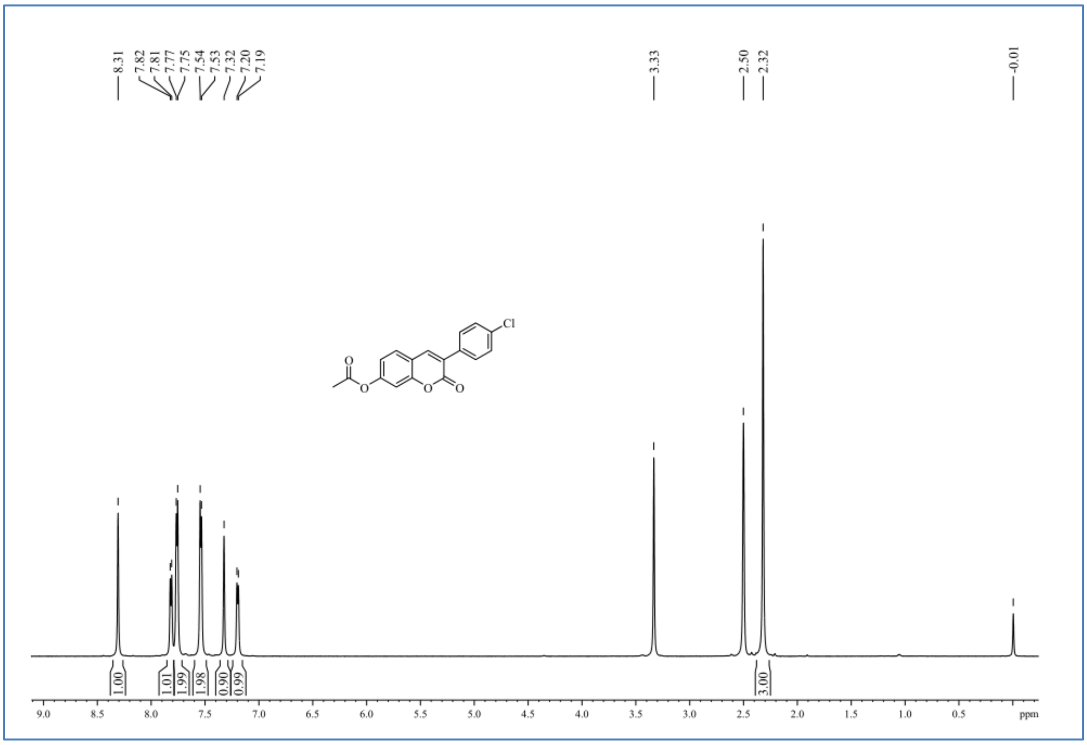

Figure S1. ^{1}H NMR spectrum of **CHAC** (400 MHz, DMSO-d_6)

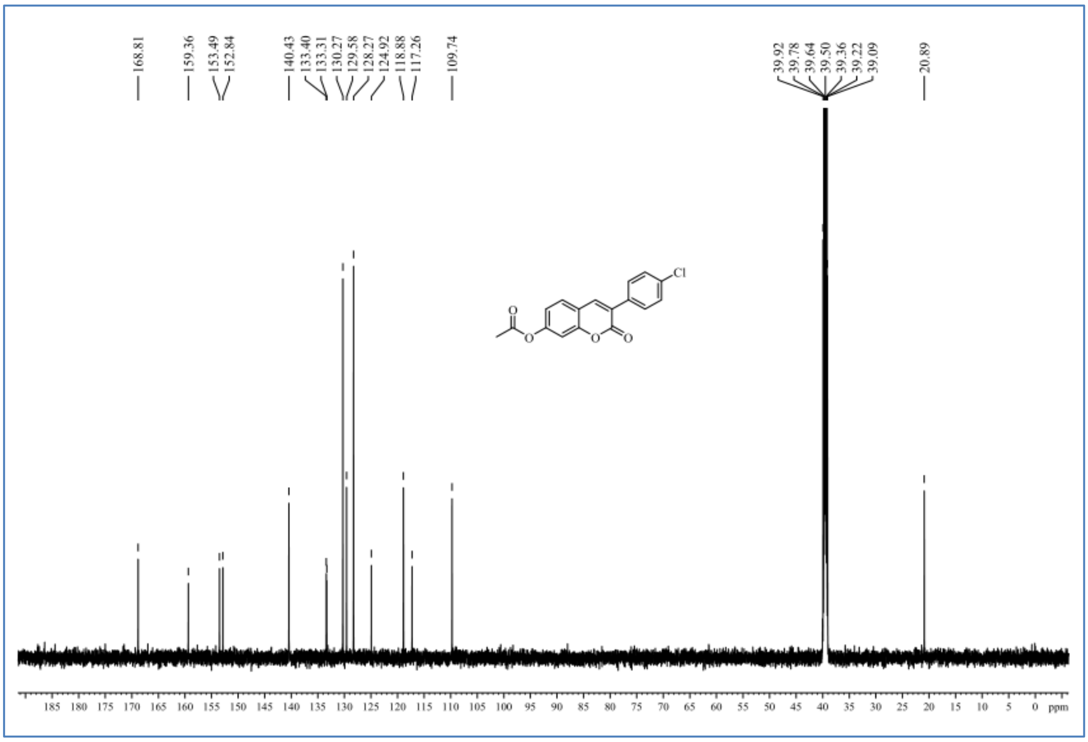

Figure S2. ^{13}C NMR spectrum of **CHAC** (400 MHz, DMSO-d_6)

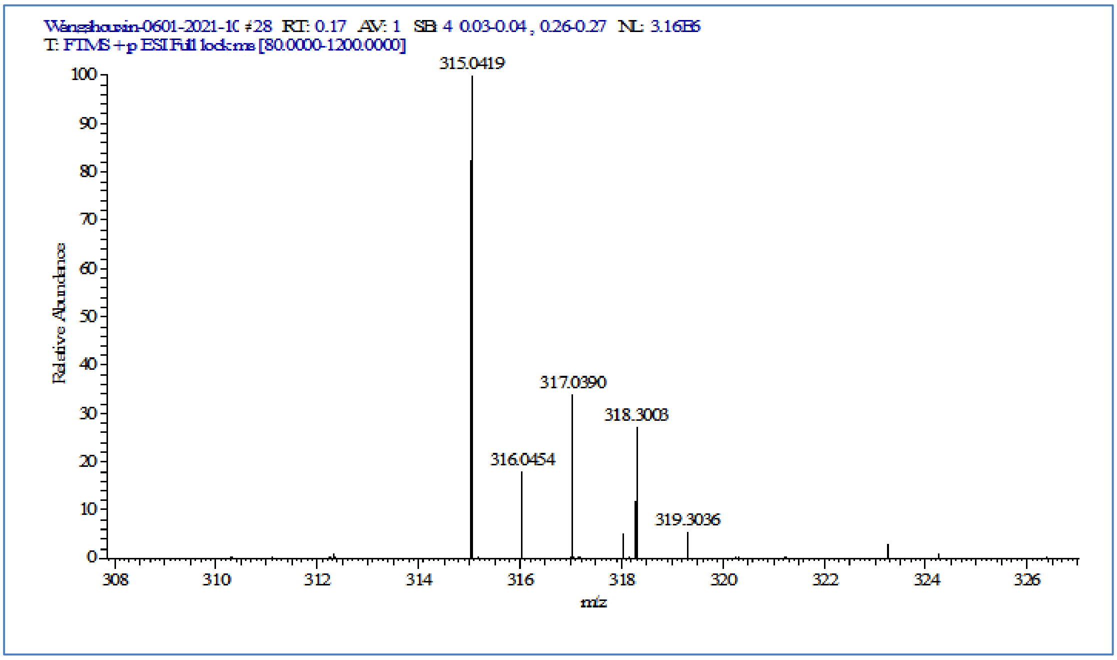

Figure S3. HRMS spectrum of the **CHAC**

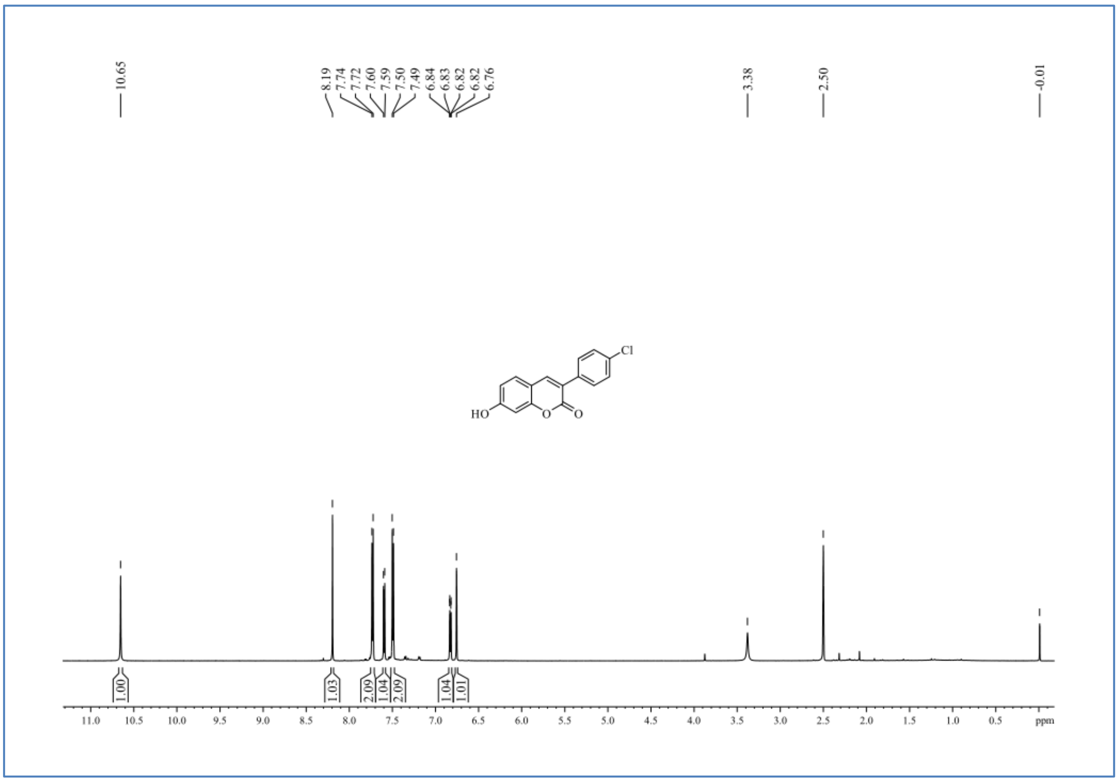

Figure S4. ^{1}H NMR spectrum of **CHOH** (400 MHz, DMSO-d_6)

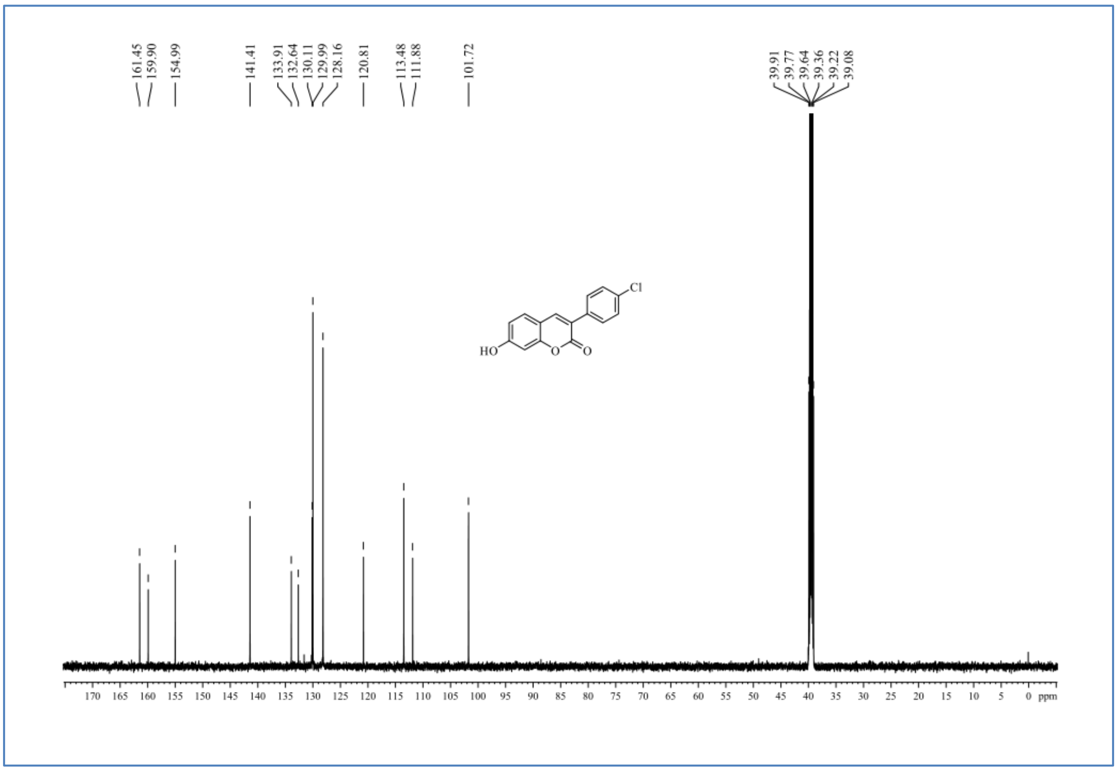

Figure S5. ^{13}C NMR spectrum of **CHOH** (400 MHz, DMSO-d_6)

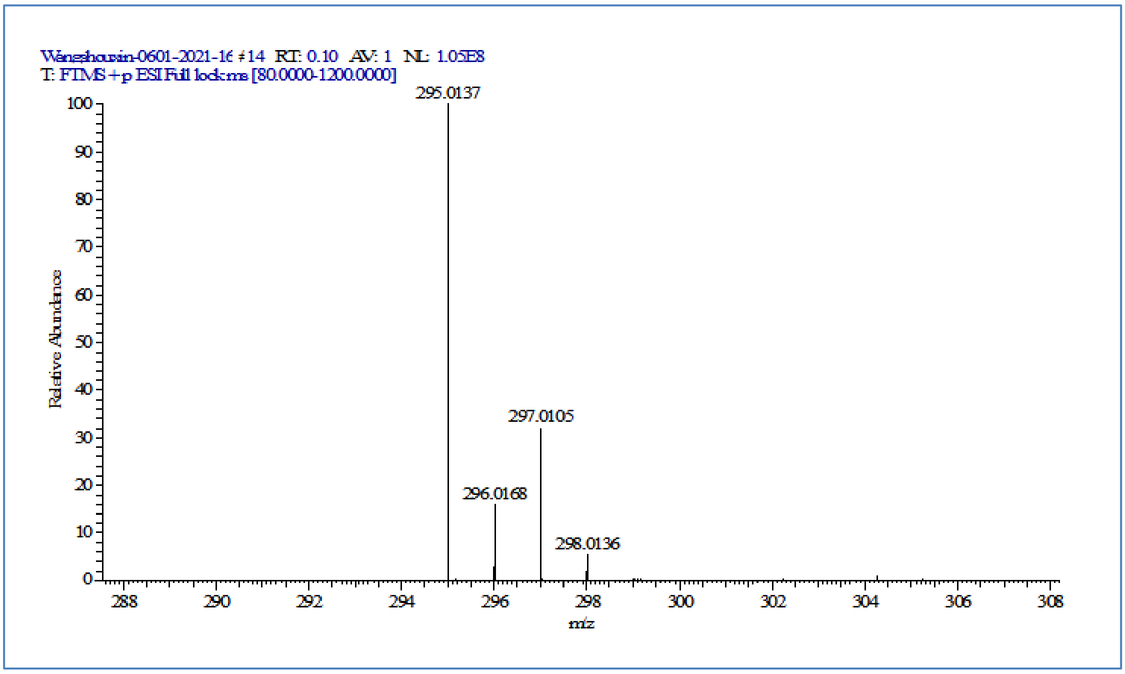

Figure S6. HRMS spectrum of **CHOH**

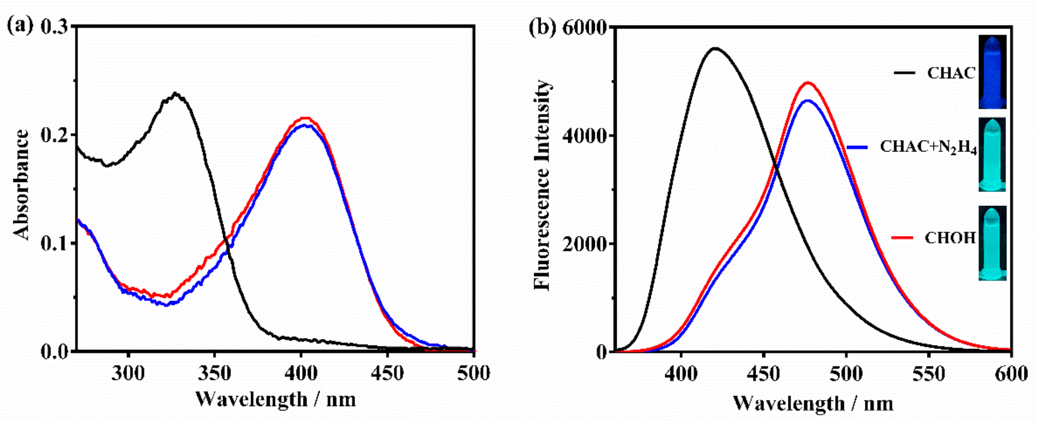

Figure S7. UV–Vis absorption (a) and fluorescence emission (b) spectra of **CHOH** (10 μM and 5 μM), and **CHAC** (10 μM and 5 μM) in piror and post reacting with N_2H_4 (100 equiv) in DMSO:PBS buffer (10 mM, 4:6 v/v, pH = 7.4), λ_{ex}= 340 nm. The inset of (b) shows photographs of **CHAC** with or without hydrazine and **CHOH** under UV light.

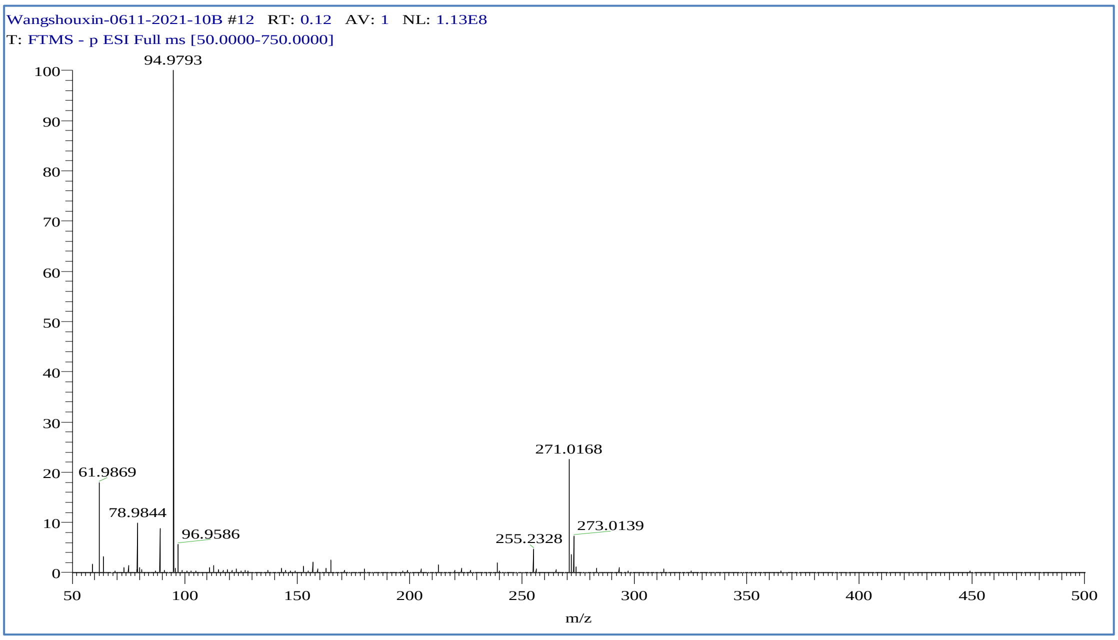

Figure S8. HRMS spectrum of **CHAC** after reaction with N_2H_4

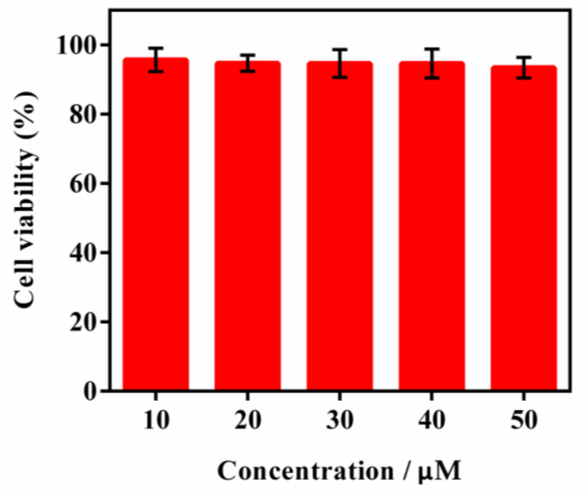

Figure S9. Various **CHAC** concentrations effects upon MC3T3-E1 cell viability

Table S1. Comparison regarding **CHAC** and other probes for N_2H_4

Probes	Type	LOD /μM	Linearity range/ μM	Effective pH range	Applications	Ref.
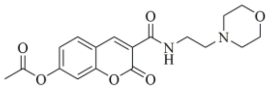	Off-On	3.93	20–70	5.5-11	Living cells	[S1]
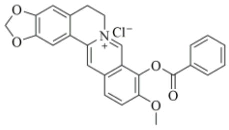	On-Off	1.37	1–350	N/A	Vapor	[S2]
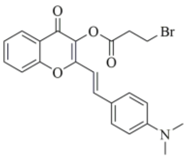	On-Off	0.36	0–20	4-8.5	Water samples	[S3]
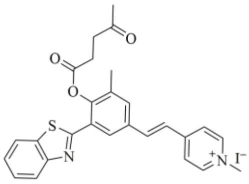	Off-On	5.47	0–210	6-11	Water samples and living cells	[S4]
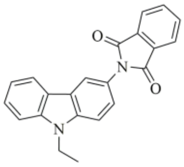	Off-On	2.67	100–400	N/A	Water samples	[S5]

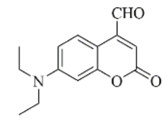	On-Off	0.022	0–1	3-11	Red wines and water samples	[S6]
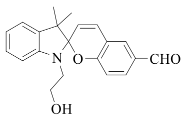	On-Off	1.26	5-100	4-10	water samples	[S7]
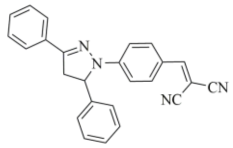	Off-On	6.16	15–35	5-8	Water samples	[S8]
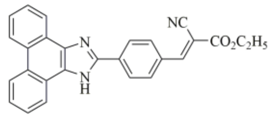	Ratiometric	1.60	0–120	6-12	Living cells	[S9]
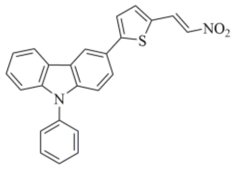	Ratiometric	0.6	0–130	5-9	Water samples and living cells	[S10]
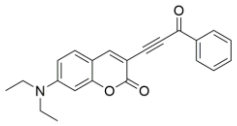	Off-On	0.267	0–350	6-10	Water samples and living cells	[S11]
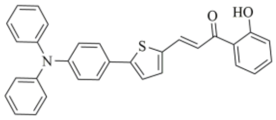	Ratiometric	0.23	0–80	6-9	Living cells	[S12]
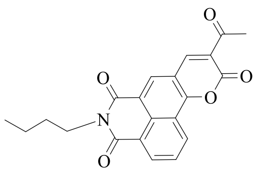	Ratiometric	0.203	60-160	6-9	vapor and living cells	[S13]
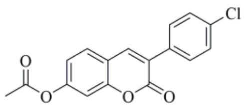	Ratiometric	0.16	0–500	6-12	Water samples, vapor, soils and living cells	This work

N/A indicates that the data is not mentioned in the reference.

References

S1. Jiang, J.-H.; Zhang, Z.-H.; Qu, J.; Wang, J.-Y. A lysosomal targeted fluorescent probe based on coumarin for monitoring hydrazine in living cells with high performance. *Anal. Methods* **2021**, *14*, 17–21.

S2. Ruan, S.; Gao, Y.; Wang, Y.; Li, M.; Yang, H.; Song, J.; Wang, Z.; Wang, S. A novel berberine-based colorimetric and fluorimetric probe for hydrazine detection. *New J. Chem*. **2020**, *44*, 15752–15757.

S3. Zhang, X.; Shi, C.; Ji, P.; Jin, X.; Liu, J.; Zhu, H. A red-emitting fluorescent probe based on flavone for hydrazine detection and its application in aqueous solution. *Anal. Methods* **2016**, *8*, 2267–2273.

S4. Tang, L.; Zhou, L.; Liu, A.; Yan, X.; Zhong, K.; Liu, X.; Gao, X.; Li, J. A new cascade reaction-based colorimetric and fluorescence "turn on" dual-function probe for cyanide and hydrazine detection. *Dyes Pigm.* **2021**, *186*, 109034.

S5. Wang, W.-D; Hu, Y.; Li, Q.; Hu, S.-L. A carbazole-based turn-on fluorescent probe for the detection of hydrazine in aqueous solution. *Inorg. Chim.* Acta. **2018**, *477*, 206–211.

S6. Wang, J.; Wang, H.; Yang, S.; Tian, H.; Liu, Y.; Hao, Y.; Zhang, J.; Sun, B. A fluorescent probe for sensitive detection of hydrazine and its application in red wine and water. *Anal. Sci.* **2018**, *34*, 329–333.

S7. Huang, X.; Zhou, Z.; Xiao, X.; Xia, L.; Li, G. Aldehyde spiropyran fluorescent probe for rapid determination of hydrazine in environmental water. *Luminescence* **2022**, *37*, 1891-1898.

S8. Zheng, X.-X.; Wang, S.-Q.; Wang, H.-Y.; Zhang, R.-R.; Liu, J.-T.; Zhao, B.-X. Novel pyrazoline-based selective fluorescent probe for the detection of hydrazine. *Spectrochim. Acta A* **2015**, *138*, 247–251.

S9. Li, Z.; Zhang, W.; Liu, C.; Yu, M.; Zhang, H.; Guo, L.; Wei, L. A colorimetric and ratiometric fluorescent probe for hydrazine and its application in living cells with low dark toxicity. *Sens. Actuators B* **2017**, *241*, 665–671.

S10. Wang, L.; Pan, Q.; Chen, Y.; Ou, Y.; Li, H.; Li, B. A dual-response ratiometric fluorescent probe for hypochlorite and hydrazine detection and its imaging in living cells. *Spectrochim. Acta A* **2020**, *241*, 118672.

S11. Wang, M.; Wang, X.; Li, X.; Yang, Z.; Guo, Z.; Zhang, J.; Ma, J.; Wei, C. A coumarin-fused 'off-on' fluorescent probe for highly selective detection of hydrazine. *Spectrochim. Acta A* **2020**, *230*, 118075.

S12. Liu, Z.; Yang, Z.; Chen, S.; Liu, Y.; Sheng, L.; Tian, Z.; Huang, D.; Xu, H. A smart reaction-based fluorescence probe for ratio detection of hydrazine and its application in living cells. *Microchem. J.* **2020**, *156*, 104809.

S13. Shi, X.; Yin, C.; Zhang, Y.; Wen, Y.; Huo F. A novel ratiometric and colorimetric fluorescent probe for hydrazine based on ring-opening reaction and its applications. *Sens. Actuators B* **2019**, *285*, 368–374.
